# Characterization of Quaternary-Ammonium-Based Ionogel Membranes for Application in Proton Exchange Membrane Fuel Cells

**DOI:** 10.3390/gels10050308

**Published:** 2024-05-01

**Authors:** Eduardo Iniesta-López, Adrián Hernández-Fernández, Ángel Martínez-López, Yolanda Garrido, Antonia Pérez de los Ríos, Francisco José Hernández-Fernández

**Affiliations:** Department of Chemical Engineering, Faculty of Chemistry, University of Murcia (UM), Campus de Espinardo, E-30100 Murcia, Spain; eduardo.iniestal@um.es (E.I.-L.); adrian.h.f@um.es (A.H.-F.); ygh46006@um.es (Y.G.); aprios@um.es (A.P.d.l.R.)

**Keywords:** ionogel, ionic liquids, fuel cell, proton exchange membrane, polymer inclusion membrane, quaternary ammonium salt, poly(vinyl chloride), ion exchange capacity, water uptake

## Abstract

In recent years, the quest to advance fuel cell technologies has intensified, driven by the imperative to reduce reliance on hydrocarbon-derived fuels and mitigate pollutant emissions. Proton exchange membranes are a critical material of fuel cell technologies. The potential of ionic liquid-based polymer inclusion membranes or ionogels for proton exchange membrane fuel cells (PEMFCs) has recently appeared. Thermal stability, SEM-EDX characterization, NMR and IR characterization, thermogravimetric analysis, ion exchange capacity, and water uptake are key properties of these membranes which need to be investigated. In this work, ionogel based on quaternary ammonium salts, such as [N_8,8,8,1_^+^][Cl^−^], [N_8,8,8,1_^+^][Br^−^], and [N_8-10,8-10,8-10,1_^+^][Cl^−^] in various compositions with poly(vinyl chloride) are extensively studied and characterized based on those key properties. The best properties were obtained when a quaternary ammonium cation was combined with a bromide anion. Finally, ionogels are tested in microbial fuel cells. Microbial fuel cells based on the ionogel reach a maximum of 147 mW/m^2^, which represents 55% of the reference membrane (Nafion 212). These results indicate that we still have the possibility of improvement through the appropriate selection of the cation and anion of the ionic liquid. Overall, the promise of ionogel membranes as a viable alternative in fuel cell applications has been demonstrated.

## 1. Introduction

Recently, considerable effort has been invested in the improvement of fuel cells with the purpose of reducing dependence on hydrocarbon-derived fuels and minimizing pollutant emissions during their operation, a situation that could pose a significant threat to the environmental and human health. One of the most noteworthy advances is fuel cells which use green and renewable fuels. These include microbial fuel cells and hydrogen fuel cells.

Most renewable energy sources are intermittent, as their production depends on other factors such as the energy market or weather conditions. It is imperative to address this mismatch between renewable energy production and market demand through suitable storage, conversion, and generation systems [1]. Utilizing renewable energy to produce green hydrogen as an energy storage vector, and employing fuel cells to convert it on demand, can help to overcome some of the key barriers of renewable energies. Microbial fuel cells (MFCs) are devices that utilize microorganisms to biodegrade organic matter in waste. Such cells have been considered a potential alternative to those utilizing fossil fuels [2,3].

Polymer electrolyte membrane fuel cells (PEMFCs) stand out as the most promising due to their modularity [4] and wide range of application. Within PEMFCs, the proton exchange membrane (PEM) plays a central and crucial role. It is essential that the PEM exhibits high proton conductivity, low electronic conductivity, thermal stability, low fuel permeability, solid mechanical properties, and affordable cost [5,6].

Currently, the most used polymer electrolyte in PEMFCs is based on expensive perfluorinated membranes, such as Nafion, which exhibit a high proton conductivity, good mechanical strength, and excellent stability. However, these membranes have several drawbacks, as to ensure good proton conduction, they must be fully hydrated, limiting their operating temperature range to below 80 °C and 100% relative humidity [7,8]. There are hydrophilic clusters in PFSA, which are sulfonic acid groups. Phase separation of the hydrophobic PTFE skeleton and hydrophilic sulfonic acid groups will occur only when the hydration is sufficient, thereby ionizing enough protons as active sites to improve the proton conductivity of the membrane [9], while for the temperature range below 80 °C, it should be related to the thermal stability issue of -SO_3_H groups. It is reported that the thermal event observed in the region between 90 °C and 250 °C is associated with the loss of the -SO_3_H groups [10]. That is why the operating temperature range is generally limited and should always be below 80 °C.

Different types of perfluorinated composite polymer membranes, functionalized non-fluorinated polymers, related compounds, and acid–base composite membranes have been investigated [11]. Very recently, new polymer inclusion membranes based on ionic liquids or ionogels have been used as proton exchange membranes in microbial fuel cells [12]. Ionic liquids (ILs) are organic salt compounds which melt at temperatures below 100 °C and generally consist of an inorganic anion and an organic cation. Ionic liquids have garnered significant attention due to their special properties, such as near-zero vapor pressure, long-term stability, and high conductivity. Further, the properties of these salts may be modified by varying the structure of the anode and cathode. ILs are considered an eco-friendly alternative to conventional organic solvents [13]. Ionogels have been employed as separators in single-chamber microbial fuel cells (MFCs) fed with different wastewaters such us slurry wastewater [14,15]. Such compositions have shown promising results in MFCs as proton exchange membranes.

To investigate the potential viability of these ionogels in PEMFCs, a comprehensive characterization is imperative. Numerous tests may be applied to evaluate properties and performance, including thermogravimetry (TG) [16], which is used to examine the relationship between temperature and the mass of a sample during a thermal process, assessing the variation in sample mass as a function of temperature changes; differential scanning calorimetry (DSC) [17], a technique that provides significant information about the thermochemical properties and stability of materials used in PEM membranes; dilatometry [18], used to measure changes in the dimensions of a sample in response to temperature variations, particularly useful for studying membrane expansion or contraction, providing valuable information on thermomechanical properties, thermal stability, and phase transitions; scanning electron microscopy (SEM), which provides detailed information about the morphology and surface structure of the membranes; energy dispersive X-ray spectroscopy (EDX), which provides information about the elemental composition and distribution of elements in the membrane, crucial for understanding chemical structure and evaluating both its quality and performance; water absorption, which emerges as a key element to maintain effectiveness in both ionic conductivity and the effective performance of the fuel cell, ensuring proper hydration of the membrane and allowing the dissociation of functional groups in the polymer, facilitating proton flow through the membrane; and ion exchange capacity determination, involving conducting an assessment to quantify the number of fixed ionic groups present in a membrane per unit weight, under specific conditions.

Some chemical and physical properties of ionogels which may be compared with those of perfluorated conventional membranes have been analyzed. Specifically, thermal stability, SEM-EDX characterization, NMR and IR characterization, thermogravimetric analysis, ion exchange capacity, and water uptake have been systematically analyzed and studied for both types of membranes. Furthermore, the ionogel membranes have been applied as proton exchange membranes in microbial fuel cell.

## 2. Results and Discussion

### 2.1. Thermal Stability of the Ionogel

**Figure 1:**
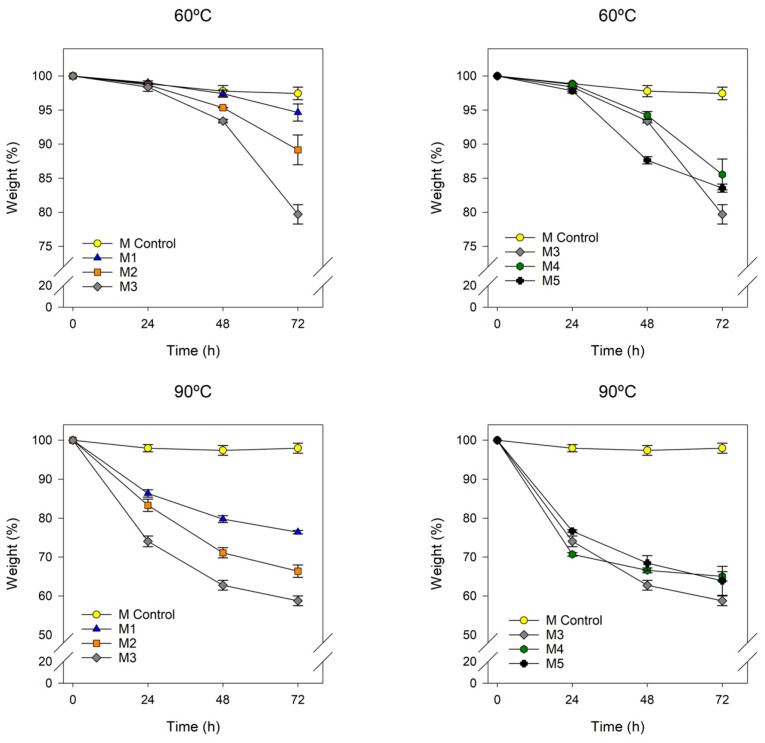
Evolution of weight loss over time for M1, M2, and M3, based on [N_8,8,8,1_^+^][Cl^−^], with IL concentration of 50%, 60% and 70%, respectively; M4 and M5, based on [N_8-10,8-10,8-10,1_^+^][Cl^−^] and [N_8,8,8,1_^+^][Br^−^] at 70% of IL and 30% of PVC, respectively; and M Control as Nafion 212.

Thermal stability analysis was carried out at two temperatures as commented in the Section 4. In Figure 1, it can be observed that, for both analyzed temperatures, the different samples based on ionic liquids exhibit similar behaviors in terms of mass loss. It can be inferred that the increase in the concentration of the ionic liquid leads to an increase in mass loss, following the sequence M3 > M2 > M1, with significant differences detected by statistical analysis. The ionogel can absorb water in its structure, and, therefore, an increase in the amount of ionic liquid results in greater mass losses. Compared to Nafion membranes, all samples show a greater weight loss as the temperature increases due to the water release from ionic liquids, with the loss being more pronounced at 90 °C.

On the other hand, in the study of different ionic liquid composition, it is observed that the membrane M4 exhibits the most favorable results compared to Nafion. The presence of longer alkyl chain groups can increase its hydrophobicity, resulting in less water retention [19]. Similarly, a bromide anion, due to its larger atomic radius, can occupy more space in the atomic structure and, therefore, exhibit higher hydrophobicity. Consequently, for both analyzed temperatures, the following sequence is verified: M4 < M5 < M3, with mass loss being more pronounced at higher temperatures.

Membranes based on ionogels exhibit significant mass losses at high temperatures due to water absorption into the membrane.

### 2.2. SEM-EDX Characterization

**Figure 2:**
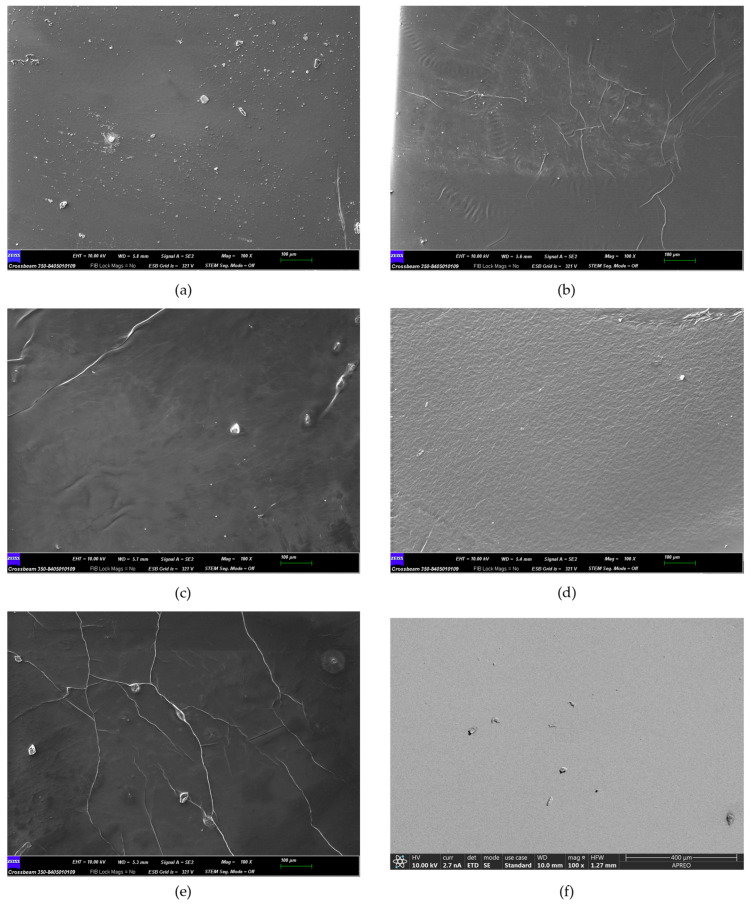
Scanning electron micrographs of different membranes: (**a**) M1: 50/50% [N_8,8,8,1_^+^][Cl^−^]/PVC; (**b**) M2: 60/40% [N_8,8,8,1_^+^][Cl^−^]/PVC; (**c**) M3: 70/30% [N_8,8,8,1_^+^][Cl^−^]/PVC; (**d**) M4: 70/30% [N_8-10,8-10,8-10,1_^+^][Cl^−^]/PVC (**e**) M5: 70/30% [N_8,8,8,1_^+^][Br^−^]/PVC; (**f**) Nafion 212.

Membranes were characterized using SEM-EDX before, after 72 h at 60 °C, and after 72 h at 90 °C. Figure 2 shows the SEM micrographs corresponding to the initial state of the membranes.

Figure 2 shows a relatively homogeneous surface, with some roughness and grooves. No significant differences between ionogels were observed in SEM micrograph. Table 1 shows the results of the EDX technique applied to the surface of the different membranes to conduct a semi-quantitative assessment of potential membrane transformations resulting from temperature variations.

EDX spectra were obtained from a sample with a thickness/depth of only a few micrometers. which means that the analysis pertains to the surface membrane. Furthermore, it should be considered that EDX is a semi-quantitative technique; however, it is a very powerful technique that provides important information. The EDX spectra of the membrane M1 (50/50% [N_8,8,8,1_^+^][Cl^−^]/PVC), M2 (60/40% [N_8,8,8,1_^+^][Cl^−^]/PVC), M3 (70/30% [N_8,8,8,1_^+^][Cl^−^]/PVC), and M4 (70/30% [N_8-10,8-10,8-10,1_^+^][Cl^−^]/PVC) showed the characteristic peaks of their elements, C K, N K, O K, and Cl K. Hydrogen is very light and it does not appear in EDX spectra. In the case of M5 (70/30% [N_8,8,8,1_^+^][Br^−^]/PVC), Br L and F K peaks also appear for Nafion 212. Other small peaks that are not characteristic of these membranes appear, such as Mg K, Si K, or Ca K, which may be due to impurities or to the fact that this is a semi-qualitative technique.

The distribution of the ionic liquid in the membrane was studied by considering the mass percentage composition of the molecular formula [N_8,8,8,1_^+^][Cl^−^](≅[N_8-10,8-10,8-10,1_^+^][Cl^−^]) which is approximately 74.3% C, 13.5% H, 8.8% Cl, and 3.5% N and PVC which comprises 38.5% C, 4.8% H, and 56.8% Cl. As can be seen in Table 1, an increase in temperature involves an increase in the C K peak and a decrease in the Cl K peak for M1. This observation suggests that as the temperature rises, ionic liquids are released from the ionogel matrix, resulting in a higher concentration of the ionic liquid on the ionogel surface. This effect is not evident in the case of ionogel with a high ionic liquid concentration (70%) where the concentration of the ionic liquids is more equilibrate across the membrane (M3 and M4). This effect is again observed in ionogel M5 (70/30% [N_8,8,8,1_^+^][Br^−^]/PVC), maybe due to the lower viscosity of [N_8,8,8,1_^+^][Br^−^] over [N_8,8,8,1_^+^][Cl^−^]. Regarding the Nafion spectrum, distinctive peaks are evident, aligning with the molecular formula of Nafion.

### 2.3. Surface Morphology and Cross-Sectional Images

**Figure 3:**
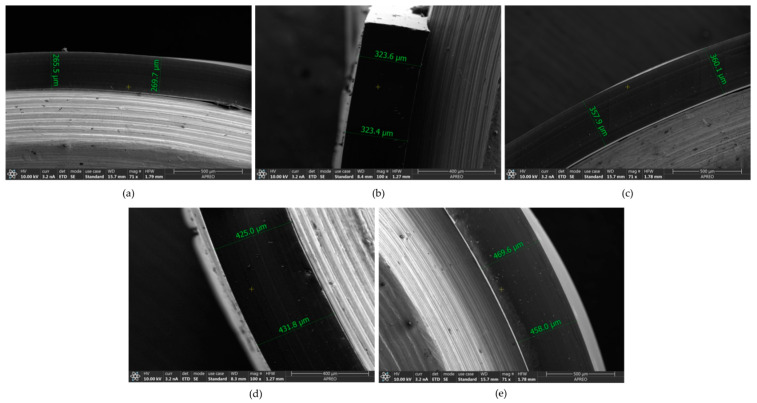
FESEM images of thickness measurement in cross-sectional membrane of 70/30% [N_8-10,8-10,8-10,1_^+^][Cl^−^]/PVC with a scale factor of: (**a**) 0.70; (**b**) 0.85; (**c**) 1; (**d**) 1.15; (**e**) 1.30.

The thickness of proton exchange membranes (PEM) in proton exchange membrane fuel cells (PEMFCs) emerges as a critical parameter significantly influencing the inherent performance and efficiency of these electrochemical systems. The optimal membrane thickness plays a fundamental role in enhancing ionic conductivity, mechanical resistance, and system durability. Inadequate thickness can negatively impact proton transport efficiency through the membrane, resulting in considerable performance losses and a shortened fuel cell lifespan. Therefore, a detailed understanding and optimization of PEM membrane thickness are presented as crucial aspects to advance the long-term effectiveness and viability of PEMFCs, opening up new avenues in the research and development of clean energy technologies. For this purpose, a methodology has been developed to define the design thickness in PEM ionogel membranes by fabricating different membranes with varying initial masses and determining their thickness by field emission scanning electron microscopy (FESEM) [20]. The prepared membranes are as follows in Table 2. Thickness was measured by FESEM (Figure 3) and are presented in Table 3. 

Statistical analysis of the data obtained led to the conclusion that there were significant differences (*p* < 0.001) between the ionogel membranes in the thickness obtained for each of the scales.

**Figure 4:**
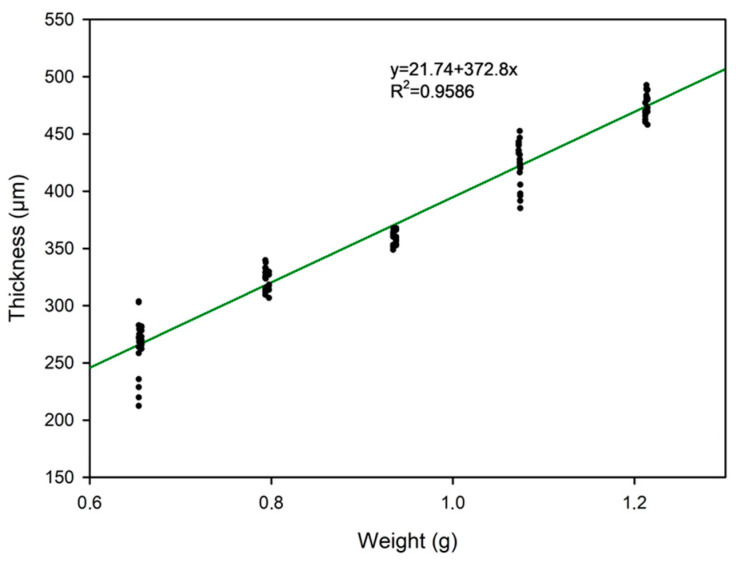
Thickness measurements obtained vs. initial mass used and the linear regression line. R^2^: correlation coefficient of the regression analysis for the experimental data.

With the obtained results, a linear regression adjustment has been performed with the aim of establishing a relationship between thickness and mass (Figure 4).

The design equation for ionogel membranes may be derived using the following equation:

(1) $$y=21.74\pm 6.72+372.8\pm 7.07\times x;R^{2}=0.9586$$

where the design equation for ionogel membranes may be derived using the following equation:

(2) $$T=\left[21.74+372.8\times W\right]\pm \sqrt{6.72^{2}+{\left[W\times 7.07\right]}^{2}}$$

where T (µm) is the membrane thickness in micrometers to be designed, and W (g) is the starting mass of the membrane in grams with corresponding error propagation.

### 2.4. Thermogravimetric Analysis (TGA)

**Figure 5:**
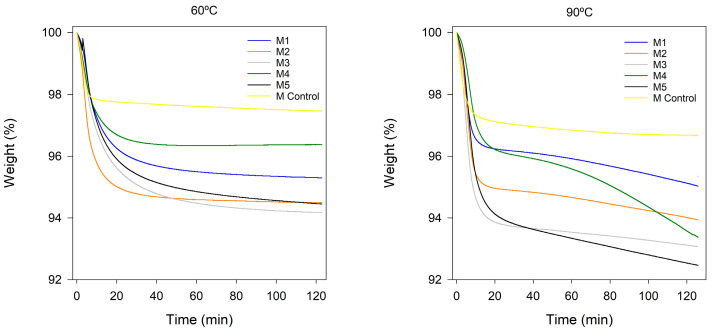
Thermogravimetric curves in isothermal mode for M1, M2, and M3, based on [N_8,8,8,1_^+^][Cl^−^] with IL concentration of 50%, 60% and 70%, respectively; M4 and M5, based on [N_8-10,8-10,8-10,1_^+^][Cl^−^] and [N_8,8,8,1_^+^][Br^−^] at 70% of IL and 30% of PVC; and M Control as Nafion 212.

**Figure 6:**
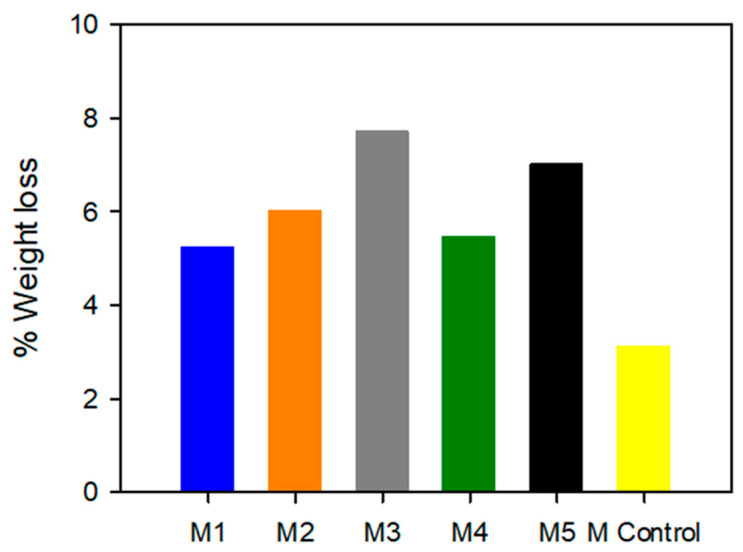
Percentage of weight loss at 150 °C for M1, M2, and M3, based on [N_8,8,8,1_^+^][Cl^−^] with IL concentration of 50%, 60% and 70%, respectively; M4 and M5, based on [N_8-10,8-10,8-10,1_^+^][Cl^−^] and [N_8,8,8,1_^+^][Br^−^] at 70% of IL and 30% of PVC; and 250 °C for M Control as Nafion 212 related to water retention.

**Figure 7:**
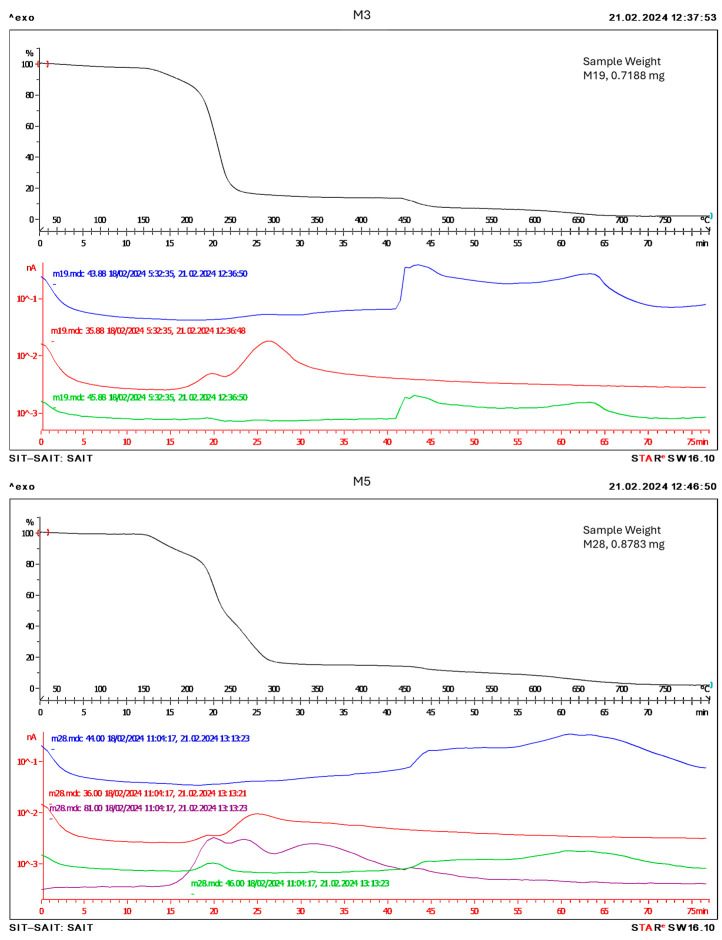
Thermogravimetric analysis coupled with mass spectrometry for M3: 70/30% [N_8,8,8,1_^+^][Cl^−^]/PVC and M5: 70/30% [N_8,8,8,1_^+^][Br^−^]/PVC. Blue line, 44 molecular weight, corresponds to CO_2_; red line, 36 molecular weight, corresponds to HCl; green line, 46 molecular weight, corresponds to NO_2_ and purple line, 81 molecular weight, corresponds to HBr.

**Figure A1:**
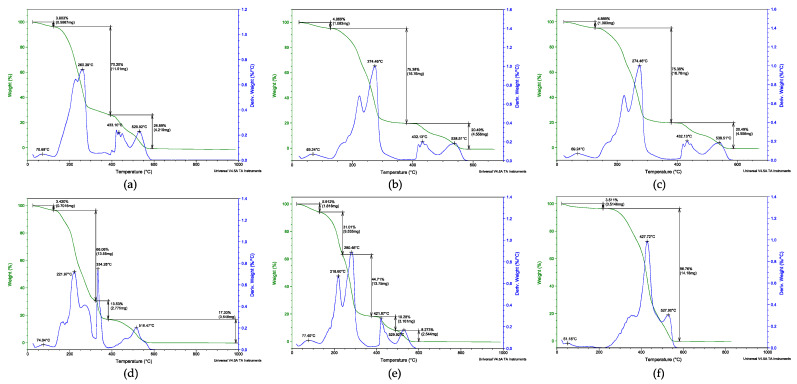
Thermogravimetric analysis in ramp temperature mode for: (**a**) M1: 50/50% [N_8,8,8,1_+][Cl^−^]/PVC; (**b**) M2: 60/40% [N_8,8,8,1_^+^][Cl^−^]/PVC; (**c**) M3: 70/30% [N_8,8,8,1_^+^][Cl^−^]/PVC; (**d**) M4: 70/30% [N_8-10,8-10,8-10,1_^+^][Cl^−^]/PVC (**e**) M5: 70/30% [N_8,8,8,1_^+^][Br^−^]/PVC; (**f**) Nafion 212. Green color represets weight (%) and blue color represents the variation of weight.

**Figure A2:**
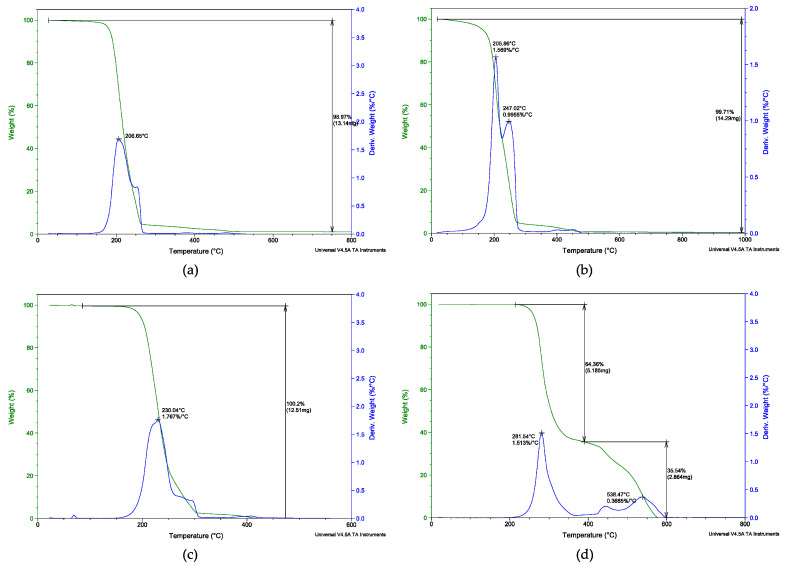
Thermogravimetric analysis in ramp temperature mode for: (**a**) M3: 70/30% [N_8,8,8,1_^+^][Cl^−^]/PVC; (**b**) M4: 70/30% [N_8-10,8-10,8-10,1_^+^][Cl^−^]/PVC (**c**) M5: 70/30% [N_8,8,8,1_^+^][Br^−^]/PVC; (**d**) Nafion 212. Green color represets weight (%) and blue color represents the variation of weight.

The thermal stability of various materials was investigated using thermogravimetric analysis (TGA) in two distinct modes: isothermal (Figure 5 and Figure 6) and temperature ramp (Figure 7, Figure A1 and Figure A2). In the isothermal method, two different temperatures were chosen to assess the membranes for various applications in hydrogen proton exchange membrane fuel cells (PEMFCs). The selected temperatures were 60 °C to evaluate their suitability for low-performance portable applications and 90 °C for more demanding applications [21]. 

Figure 5 illustrates some general trends on the loss of water from the membrane. An increase in the concentration of ionic liquid in the membrane resulted in an elevation in weight loss at both temperatures, since as the amount of ionic liquid in the membrane increases, the amount of water retained in it also increases. The increase in the alkyl chain length of the cation involves less water retention and, consequently, reduced water losses, since a longer alkyl chain length increases hydrophobicity and reduces water absorption. The ionic liquids based on bromide (M_5_) lose less water than those based on chloride (M_3_), which could be explained by the reduced electronegativity of bromide which involves less water retention. 

It is noted that membranes based on persulfonic acids exhibit less weight loss since less water is retained in their structure in the isotherm assays.

The complete degradation of the materials has been determined using a temperature ramp in thermogravimetric analysis. The decomposition of the membranes occurred in distinct stages, with the initial stage involving the release of retained water, as clearly seen in the initial slope of the six thermograms presented in Figure A1 (Appendix A). For membranes based on ionic liquids, the first phase of losses occurred at temperatures below 150 °C, attributed to the presence of retained water residues [22] and residual solvent remnants. A method for determining the water content involves calculating the difference between the initial weight and the weight loss at 150 °C [23]. Figure 6 illustrates how these results are consistent with previously obtained observations in terms of water retention. Figure 6 shows similar trends than in TGA experiments at 60 °C and 90 °C. An increase in ionic liquids concentration (M1 to M3) involves an increase in water retention, and the use of a longer alkyl chain length results in a decrease in water retention (M3 and M5, respectively). Furthermore, the ionic liquid based on the bromide anion shows less water retention than the ionic liquid based on the chloride anion.

For the perfluorosulfonic acid membrane, water loss was observed up to 250 °C and was associated with the desorption of water bonded to hydrophilic sulfonic groups, which was not completely removed during the drying process. The weight loss between 250 and 440 °C was attributed to the loss of sulfonic acid (-SO_3_H) groups. Finally, the weight loss above 440 °C was linked to the decomposition of both linkages and the polymer main chain [24]. Figure A1illustrates that Nafion was completely decomposed at 550 °C.

The thermograms of the different ionogels and Nafion 212 and those of their components separately are presented in Figure A1 and Figure A2 of Appendix A. Two parameters were chosen to study the effect of temperature on the thermograms: (1) the weight (%) of the membrane at 200 °C and the highest derivative temperature (HDT (°C) (see Table 4). Among the ionic liquids analyzed, the one that contains the bromide anion is the one that maintained the most weight (93%) at 200 °C and whose higher speed of decomposition was reached at a higher temperature which could be due to its greater thermal stability, its greater hydrophobicity, and even its lower number of volatile impurities. The thermal stability of ionic liquids strongly depends on the type of anion [25]. The increase in the ionic liquid component in the [N_8,8,8,1_^+^][Cl^−^]/PVC formulation did not imply significant differences in the parameters studied in Table 4, although a minor increase in weight loss of the membranes whose formulation contained more amount of the liquid ionic phase was observed. As mentioned above, this behavior could be due to the amount of water absorbed in the ionic liquid phase. For the ionogels prepared at 70% of ionic liquid and PVC, a lower weight loss was observed for the ionogel based on the bromide ionic liquid. This behavior was related to the lesser weight loss observed when the TGA values of ionic liquids were analyzed individually and commented above. For Nafion 212, lower weight losses were observed at 200 °C and HDT was observed at approximately 470 °C.

Thermogravimetric analysis coupled with mass spectrometry (TG-EM) was carried out for all samples to understand the decomposition process. Figure 7 shows the results obtained for the ionogel membranes based on chloride and bromide.

In Figure 7, we can observe the profile of the compounds CO_2_ (blue line), HCl (red line), NO_2_ (green line), and HBr (purple line). Those are the decomposition compounds of the ionogel membranes. As can be seen in Figure 7 (M3), firstly the anion of the ionic liquids (chloride) and chloride of the PVC is decomposed. Then, the cation of the ionic liquids (NO_2_ coming from the cation) and the rest of the membrane (CO_2_) is decomposed. In Figure 7 (M5), HBr appears in the first time; however, the peak is wider which could be related to the higher stability of the membrane which contains the bromide-based ionic liquid compared with that based on chloride ionic liquid, as it was commented above. The second peak corresponds to HCl, due to the decomposition of chloride from PVC, then NO_2_ and CO_2_ from the cation of the ionic liquid and PVC, respectively. The rest of the TG-EM test are similar to the M3 sample since these samples are based on tetraalkyl ammonium cations combined with chloride anions. 

### 2.5. Ion Exchange Capacity (IEC)

The membrane is one of the key factors that determines the direction of the reaction mechanism of proton exchange membrane fuel cell (PEMFC) systems and thus the overall performance of the system [26,27]. In PEMFC systems, it is necessary to ensure efficient transport of protons so that the redox reaction that is taking place is facilitated. Permeability and ion flux through the membrane are parameters that are subject to the influence of the properties of PIMs. Thus, it is essential to develop a membrane whose materials confer suitable structure and properties in terms of ion exchange capacity and water uptake.

Both IEC and water uptake are parameters that may be related to two long-established basic proton transfer mechanisms, the vehicle mechanism and the Grotthuss mechanism [28,29]. In the first of these mechanisms, protons diffuse through the aqueous medium via a molecule known as the proton “vehicle”, a hydronium ion (H_3_O^+^), whereas in the second mechanism protons form and break hydrogen bonds, which they use to hop between water molecules.

The results obtained for the ionogel membranes tested are presented in Table 5. It could be concluded that the commercial Nafion 212 membrane exhibits the highest value overall, with an IEC average value of 0.865 mmol/g, similar to those reported in other papers [30,31]. On the other hand, IEC values for all ionogel membranes are much lower than those achieved by Nafion 212. This is because these ionogel membranes are formed by ionic liquids that do not contain protonable functional groups such as hydroxyl (-OH), carboxyl (-COOH), or amino (-NH_2_) among others, so they are only able to incorporate in their structure a very small number of protons. Although these ionogel membranes did not present a high IEC value, M.J. Salar-García et al. [32] demonstrated that the ionic liquid [N_8,8,8,1_^+^][Cl^−^], when combined with PVC in 70/30% IL/PVC ratios to form an ionogel membrane, was able to transport protons efficiently in a microbial fuel cell. This result indicates that the main proton transport mechanism is not based on ionic exchange, although this does not mean that it is not through a vehicle mechanism or Grotthuss mechanism through water microenvironments or using the anion of the ionic liquids themselves. In spite of this fact, the statistical analysis of the data obtained allowed us to conclude that between the ionogel membranes M1, M2, and M3, whose IEC values (mean values ± SD) were 0.022 ± 0.002, 0.028 ± 0.008, and 0.015 ± 0.003, respectively, there were no significant differences (*p* < 0.05). All the above-mentioned seems to indicate that IEC is not a suitable parameter to study the proton exchange capacity of the ionic liquids chosen for this study.

### 2.6. Water Uptake

Water plays an important role in PEMFC systems, it is particularly involved in the formation of hydrogen bridges between ion exchange sites, thus the water uptake of ionogel membranes is related to the Vehicle and Grotthuss proton transport mechanisms [33].

**Figure 8:**
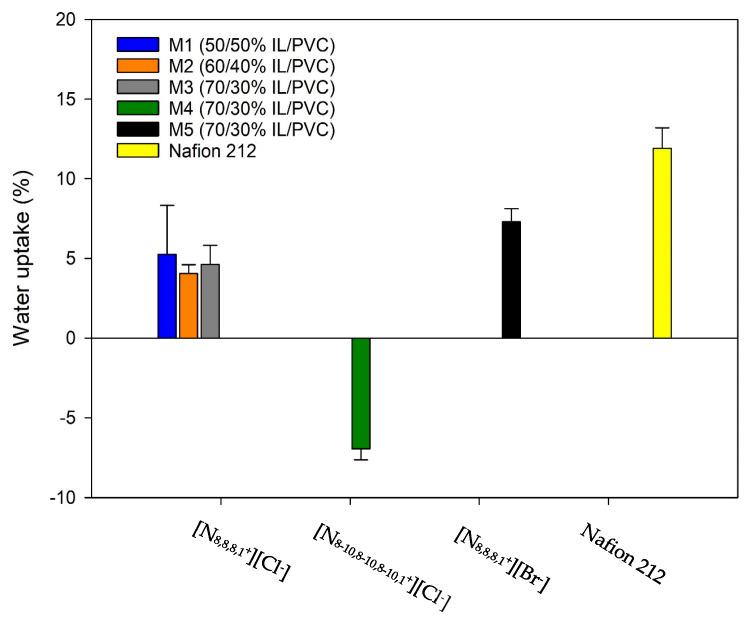
Water uptake of ionogel membranes with different amounts of ionic liquid (M1, M2, and M3), different ionic liquids (M4 and M5), and the commercial membrane Nafion 212. M1: 50/50% [N_8,8,8,1_^+^][Cl^−^]/PVC. M2: 60/40% [N_8,8,8,1_^+^][Cl^−^]/PVC. M3: 70/30% [N_8,8,8,1_^+^][Cl^−^]/PVC. M4: 70/30% [N_8-10,8-10,8-10,1_^+^][Cl^−^]/PVC. M5: 70/30% [N_8,8,8,1_^+^][Br^−^]/PVC.

Figure 8 shows that the commercial membrane Nafion 212, used as reference, presented an average water absorption value of 11.92%, which is similar to those reported elsewhere [30,34]. The membranes formed by the [N_8,8,8,1_^+^][Cl^−^] ionic liquid with different IL/PVC proportions showed positive water uptake after being submerged for 24 h, resulting in average water uptake values of 5.25%, 4.06% and 4.62% for membranes M1, M2 and M3, respectively. Despite the above, the statistical analysis indicates that there were no significant differences (*p* < 0.05) between the water uptake values obtained for membranes M1, M2 and M3, so that an increase in the amount of [N_8,8,8,1_^+^][Cl^−^] would not lead to greater water uptake in a membrane in contact with water for 24 h.

The ionogel membrane M4, formed by the ionic liquid [N_8-10,8-10,8-10,1_^+^][Cl^−^], was the only one that showed mass loss in water after 24 h, with an average water uptake value of −6.94%. It is due to this ionic liquid containing water soluble impurities (70% purity) as can be seen observed from safety data sheet Sigma-Aldrich, (Darmstadt, Germany).

Finally, the M5 membrane had an average positive water uptake value of 7.30%, which indicates that this ionogel membrane did not lose mass when in contact with water over 24 h. Furthermore, in this case the statistical analysis detected significant differences (*p* < 0.05) between the M5 membrane and M4 membrane, indicating that the anion modification in the ionic liquid could influence water uptake in an ionogel membrane with PVC as a polymer.

Despite all the above, statistical analysis indicated that for membranes with a mass of 1.88 g these small differences in mass (4–7%) were not significant (*p* < 0.05). We would be talking about increases or decreases in a few milligrams that may be overshadowed by the error of the analytical balance itself.

### 2.7. Oxidative Stability

A very widespread analysis to test the oxidative stability of proton exchange membranes used in fuel cells is the Fenton test.

**Figure 9:**
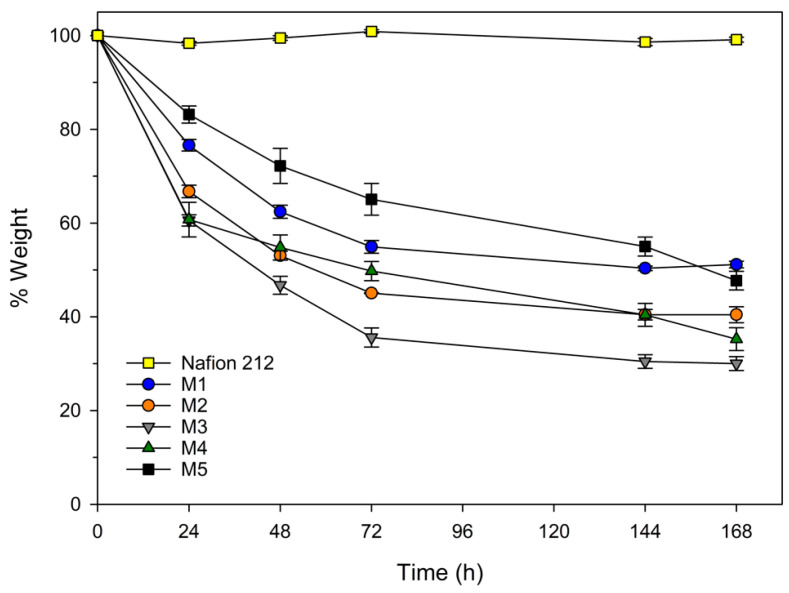
Oxidative stability of ionogel membranes with different amounts of ionic liquid (M1, M2, and M3), different ionic liquids (M4 and M5), and the commercial membrane Nafion 212 using the Fenton test at 60 °C. M1: 50/50% [N_8,8,8,1_^+^][Cl^−^]/PVC. M2: 60/40% [N_8,8,8,1_^+^][Cl^−^]/PVC. M3: 70/30% [N_8,8,8,1_^+^][Cl^−^]/PVC. M4: 70/30% [N_8-10,8-10,8-10,1_^+^][Cl^−^]/PVC. M5: 70/30% [N_8,8,8,1_^+^][Br^−^]/PVC.

The oxidative resistance of ionogel membranes based on ionic liquids [N_8,8,8,1_^+^][Cl^−^], [N_8-10,8-10,8-10,1_^+^][Cl^−^], and [N_8,8,8,1_^+^][Br^−^] after immersion in a Fenton solution of 4 ppm Fe^2+^ + 3%wt H_2_O_2_ at 60 °C is shown in Figure 9, which depicts the weight loss of these ionogel membranes in 24 h cycles.

The ionogel membranes M1, M2, and M3, which correspond to those formed by different proportions of [N_8,8,8,1_^+^][Cl^−^]/PVC, show significant differences in weight loss following the sequence M3 > M2 > M1, with M3 being the one with the highest losses. This implies that an increase in the concentration of this ionic liquid implies higher mass losses in the ionogel membrane.

The ionogel membrane M4, formed by ionic liquid [N_8-10,8-10,8-10,1_^+^][Cl^−^] and M5, formed by [N_8,8,8,1_^+^][Br^−^], which has the same IL/PVC ratio as M3, presents significant differences among themselves and with M3, which implies that modifying the ionic liquid has an effect on the resistance to chemical oxidation by the radicals, with the ionogel membrane M5 being the most stable of the three. It means that by the adequate design of the ionic liquid of membrane, the stability of the membrane in the Fenton test could be significantly improved. 

Although significant mass losses were observed in all ionogel membranes, so all the mass loss of the membrane was not due to oxidative degradation by the action of radicals, part of the mass loss was due to losses of water retained by the ionic liquids during the Fenton experiment, as already discussed in Section 2.1, or the release of ionic liquid from the PVC membrane. Moreover, in any case our membranes did not completely dissolve in the reagent or break into pieces, but rather they went from a viscous and elastic state to a more rigid and fragile state, presumably because there were greater losses of ionic liquid than polymer. Chen et al. [35] reported that their polymeric proton exchange membranes based on sulfonated fluorinated poly(arylene ether)s, despite maintaining more than 90% of their initial weight after the test, broke into pieces after 2–5 h of remaining immersed in the Fenton reagent. Similarly, Li et al. [36] reported that their membranes based on methylpyrrolidinium grafted poly (vinyl benzyl chloride) dissolved completely in the Fenton reagent after 48 h of testing, and that by adding polysulfone and N,N,N′,N′-tetramethyl-1,6-hexanediamine as crosslinker, although their oxidative stability was greatly improved, they broke into small fragments after 96 to 120 h of immersion in the Fenton reagent. The Nafion 212 membrane did not show an appreciable mass loss or rupture at any time, which agrees with results reported by other authors [35].

### 2.8. Characterization and Structure

To characterize the chemical structure of the ionogel membranes in this study, ^1^H nuclear magnetic resonance (^1^HNMR) and Fourier transform infrared (FTIR) analyses were performed to obtain the characteristic spectra of the membranes, as well as those of their pure components separately. This study was carried out for [N_8-10,8-10,8-10,1_^+^][Cl^−^] because similar results are expected for the other ionic liquid membranes.

**Figure 10:**
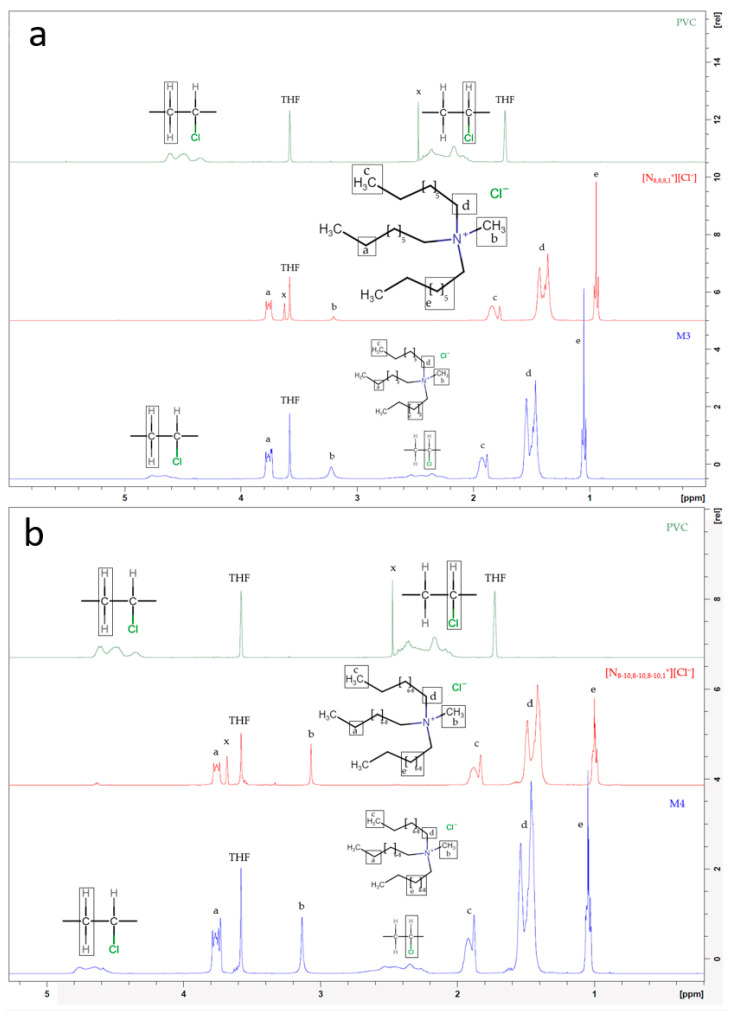
(**a**) ^1^HNMR spectra of PVC, ionic liquid [N_8,8,8,1_^+^][Cl^−^], and M3 (70/30% [N_8-10,8-10,8-10,1_^+^][Cl^−^]/PVC); and (**b**) ^1^HNMR spectra of PVC, ionic liquid [N_8-10,8-10,8-10,1_^+^][Cl^−^], and M4 (70/30% [N_8-10,8-10,8-10,1_^+^][Cl^−^]/PVC), in tetrahydrofuran. Letters (a,b,c,d,e) represent different types of carbon atoms in the ionic liquids. X represents impurities founded in the pure THF and ionic liquids.

**Figure A3:**
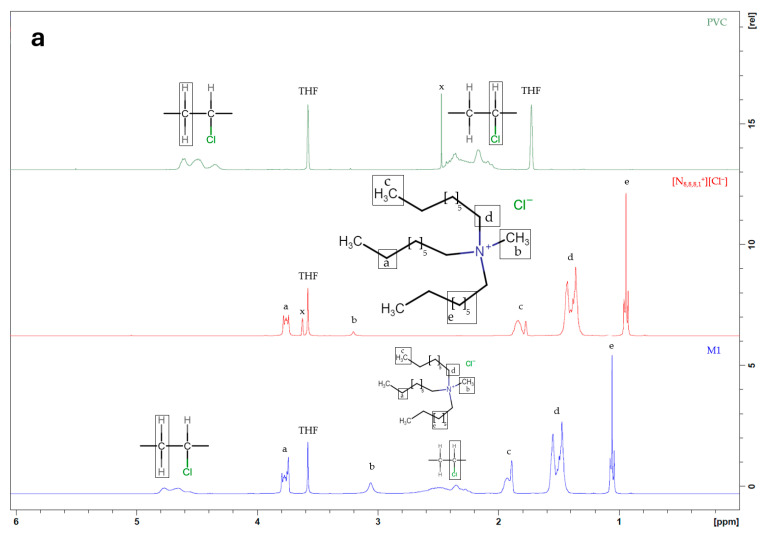

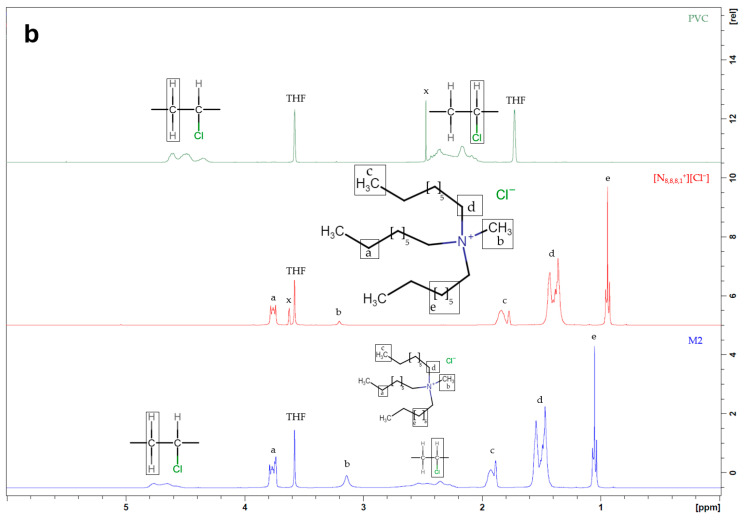
(**a**) ^1^HNMR spectra of PVC, ionic liquid [N_8,8,8,1_^+^][Cl^−^] and, M1 (50/50% [N_8,8,8,1_^+^][Cl^−^]/PVC) and, (**b**) ^1^HNMR spectra of PVC, ionic liquid [N_8,8,8,1_^+^][Cl^−^] and, M2 (60/40% [N_8,8,8,1_^+^][Cl^−^]/PVC), in tetrahydrofuran.

As can be seen in Figure 10a,b, for the PVC spectrum we would have two singlets at 1.7 ppm and 3.6 ppm that would correspond to the THF used as the solvent [37]. The singlet at 1.7 ppm disappears for [N_8-10,8-10,8-10,1_^+^][Cl^−^], [N_8,8,8,1_^+^][Cl^−^], M3, and M4 spectra, but the singlet at 3.6 ppm remains. The peaks marked with an x would correspond to impurities in the pure compounds, which subsequently do not appear on M3 and M4 spectra. The shift of protons towards higher delta values in the ionic liquid membrane relative to the individual components is the result of the interaction between the ionic liquid and the PVC of the membrane. The ^1^HNMR spectra of the ionogel membranes M1 and M2 are presented in Figure A3 of Appendix A. Similar behavior was observed for M_1_ and M_2_^1^HNMR spectra.

**Figure 11:**
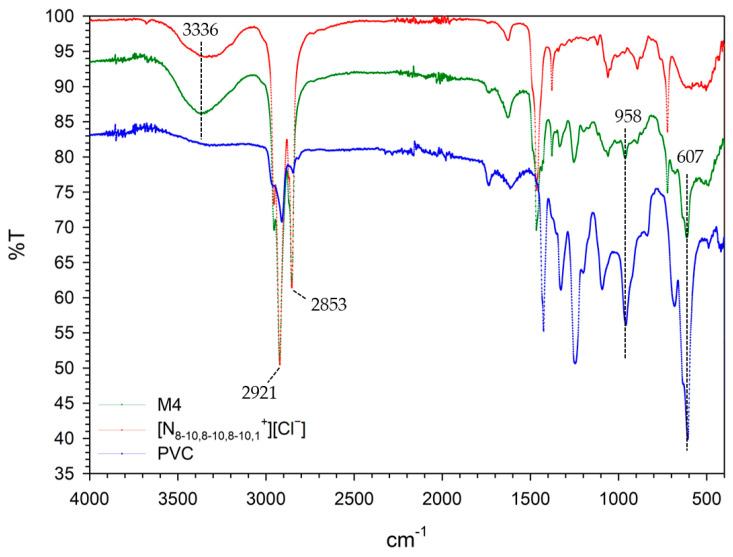
FTIR spectra of PVC, ionic liquid [N_8-10,8-10,8-10,1_^+^][Cl^−^](70/30% [N_8-10,8-10,8-10,1_^+^][Cl^−^]/PVC), and M4. Dotted lines point out the characteristic FTIR peaks.

In the infrared spectra (Figure 11), the characteristic peaks of the components separately and of the membrane appear. We can observe the characteristic peak of H_2_O at 3336 cm^−1^. It would correspond to the water absorbed by the ionic liquid and by the membrane that contains the ionic liquid. The peaks around 2900 cm^−1^ correspond to CH bonds present in all compounds. The peak at 607 cm^−1^ corresponds to the CCl bond, which is not present in the ionic liquid but is present in the PVC and in the membrane formulated with the ionic liquid. 

### 2.9. Aplication of Ionic Liquids in Microbial Fuel Cell

**Figure 12:**
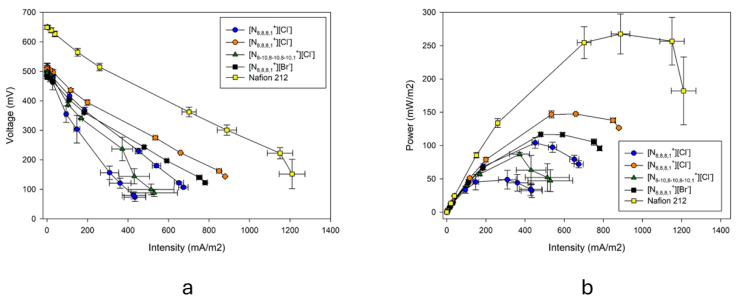
(**a**) Polarization curves and (**b**) power curves at 25 °C for M1, M2, and M3, based on [N_8,8,8,1_^+^][Cl^−^] with an IL concentration of 50%, 60%, and 70%, respectively; M4 and M5, based on [N_8-10,8-10,8-10,1_^+^][Cl^−^] and [N_8,8,8,1_^+^][Br^−^] at 70% of IL and 30% of PVC; and Nafion 212.

The use of ionic liquid-based membranes in microbial fuel cells has been studied and compared with the use of a Nafion membrane. For its study, the polarization curves of the different membranes have been determined (Figure 12) and the maximum power values have been obtained (Table 6). It has been observed that for the membranes based on 60/40% [N_8,8,8,1_^+^][Cl^−^]/PVC, up to 50% of the power achieved with Nafion can be achieved. These values are a very positive result, considering the lower cost of membranes based on ionic liquids compared to the membrane by Nafion. The lower power achieved by 70/30% [N_8-10,8-10,8-10,1_^+^][Cl^−^]/PVC membrane could be explained by the greater hydrophobicity of the immobilized ionic liquid and, consequently, its reduced water absorption. This issue has to be deeply studied. The powers of the other two membranes were around 40% of the Nafion membrane. 

The thickness of the membrane influences its proton conductivity, and, therefore, the power generated by the fuel cell. Although the thickness of the membranes based on ionic liquids tested is much greater than that of the Nafion 212 membrane, the maximum power values achieved with the ionic liquid membranes are considered good considering the ideal value reached with the Nafion 212 membrane. Reducing the thickness of membranes based on ionic liquids could reduce their ionic resistance and thereby further improve their maximum power values, bringing them even closer to the ideal values of the Nafion 212 membrane.

## 3. Conclusions

Ionogel membranes containing ionic liquid of the quaternary ammonium salts [N_8,8,8,1_^+^][Cl^−^], [N_8-10,8-10,8-10,1_^+^][Cl^−^], and [N_8,8,8,1_^+^][Br^−^], were prepared and characterized. A design equation was derived to establish a relationship between thickness and initial mass, providing a valuable tool for optimizing ionogel membranes. Regarding thermogravimetric analysis, the thermal stability of ionic liquid membranes is enough to be applied in hydrogen fuel cell application. Isothermal and temperature ramp modes revealed distinct stages of decomposition. The first lost weight is related to water absorb by the ionic liquids. The higher loss of weight is related with the ionic liquid composition. The use of bromide counter anion could improve the thermal stability and chemical stability ionogel based on thermogravimetry and Fenton studies. The membranes based on ionic liquids powers, in microbial fuel cell, range from 33% to 55% of that for Nafion in microbial fuel cell, even though the thickness of ionogel was greater. We still have room for improvement such as thermal, oxidative activity, or proton exchange. Considering that the active phase is the ionic liquid, it is possible to look for ionic liquids or mixtures of liquids that improve these properties and those could be incorporated into the ionogel. This work shows that ionogel membranes based on ionic liquids are promising in low-cost fuel cell applications. 

## 4. Materials and Methods

### 4.1. Materials

**Figure 13:**
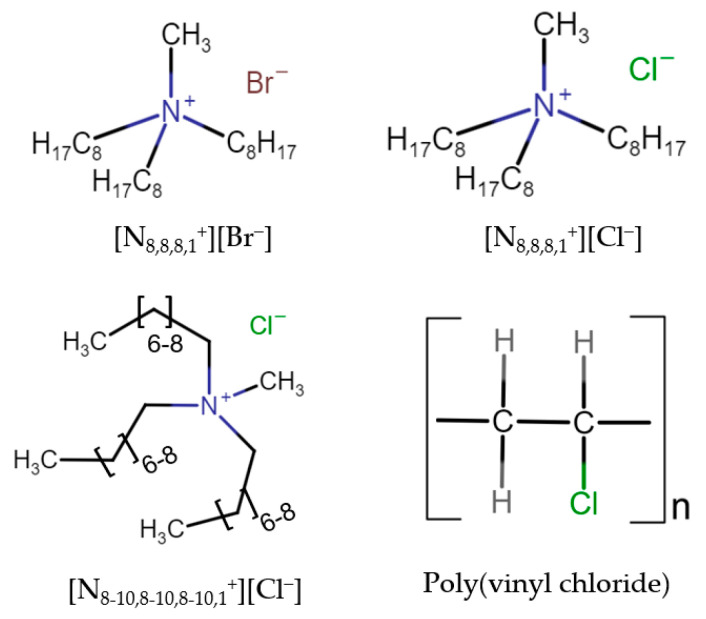
Chemical structures of ionic liquids and polymer.

The ionic liquid studied was based on quaternary ammonium cations and different anions. Specifically, methyltrioctylammonium chloride (Aliquat 336, [N_8,8,8,1_^+^][Cl^−^]), methyl trialkylammonium chloride (Aliquat, [N_8-10,8-10,8-10,1_^+^][Cl^−^]), and methyl trioctylammonium bromide ([N_8,8,8,1_^+^][Br^−^]) were acquired from Sigma-Aldrich (Darmstadt, Germany); [N_8,8,8,1_^+^][Cl^−^] and [N_8,8,8,1_^+^][Br^−^] were of a purity greater than or equal to 97%, whereas [N_8-10,8-10,8-10,1_^+^][Cl^−^] could only be purchased with a purity of 70%. The polymer poly(vinyl chloride) (PVC) was provided from Sigma Aldrich Fluka Chemical Co. (Madrid, Spain). Tetrahydrofuran (THF), hydrochloric acid (HCl), sodium chloride (NaCl), sodium hydroxide (NaOH), and oxalic acid were of reagent-grade quality and were purchased from Sigma-Aldrich (Darmstadt, Germany), Deionized water was used in all experiments. The commercial Nafion 212 PFSA DuPont membrane was obtained from H2Planet (Bizkaia, Spain). The chemical structures of the various ionic liquids and polymers employed in the preparation of ionogel membranes are presented in Figure 13.

### 4.2. Membrane Preparation

Ionogel membranes of different sizes were prepared, but always maintaining the same mass/size ratio so that the membrane thickness remained consistent, except for thickness analyses, where the size was fixed, and the masses were varied. Table 7 shows the different ionic liquid/polymer mixtures prepared, together with their total weight and size of the circular flat glass used in their preparation. The ionogel membranes were prepared following a modification of the casting method previously described by other authors [38]. Depending on which membrane we wanted to prepare, special attention was paid to the percentages of IL and PVC and the total mass of the membrane to be prepared. Taking as an example an M1 membrane intended to be analyzed by TGA, 0.2 g of ionic liquid and 0.2 g of PVC were dissolved in 3 mL of THF (15 mL for a total membrane mass of 1.88 g). The mixture was left to stir magnetically at room temperature, about 25 °C, for 20 min until a homogeneous solution was obtained. The mixture was then poured onto a circular flat glass and the organic solvent was allowed to evaporate for 12 h until the gel state was reached on the membrane. The membrane was then carefully removed from the glass and stored for further analysis.

### 4.3. Membrane Characterization

#### 4.3.1. Thermal Stability Tests

The thermogravimetric analyses (TGA) were performed with a TA Instruments TGA-DSC Simultaneous Analyzer, model SDT 2960. Two types of tests were carried out using 15 mg ionogel-based PIMs samples. The selected temperatures used for constant temperature tests were 60 °C and 90 °C, while for variable temperature tests a heating ramp of 10 °C/min was applied in a heating cycle set from 25 °C to 600 °C. All tests were conducted under an air atmosphere.

Longer duration tests were also performed for the same temperatures selected in the constant temperature tests discussed above, 60 °C and 90 °C. A 0.4 g sample of ionogel membrane was kept for 72 h at constant temperature in a Vaciotem-TV (J.P. Selecta) vacuum oven. To control the weight of the sample during the assay, the samples were removed from the oven in 24 h periods, allowed to temper in a desiccator for 1 h, and then they were weighed.

The thermogravimetric analyses (TGA) were performed with a TA Instruments TGA-DSC Simultaneous Analyzer, model SDT 2960.

In the case of thermogravimetric analysis coupled with mass spectrometry (TG-EM), the TG is coupled to a quadrupole mass spectrometer, “ThermoStar”, QMS 300M3, from Balzers, with a scanning range of 1 to 300 amu, from 0.2 to 10 s/uma. In this case, a heating ramp of 10 °C/min was applied in a heating cycle set from 30 °C to 800 °C.

#### 4.3.2. SEM-EDX Characterization

To perform a detailed analysis of the surface morphology of the different ionogel membranes, a platinum coating was applied. Examinations were then performed under vacuum using an ApreoS Field Emission Scanning Electron Microscope (FESEM). This identical instrumentation was also used to accurately determine the thickness of ionogel membranes. This procedure requires initial preparation of the ionogel membranes, which involves precise sectioning of a 1 × 1 cm segment, followed by platinum coating and finally placement on a specialized cylindrical support structure for SEM measurement.

For comprehensive evaluation of chemical composition, ionogel membranes were examined after fabrication and after evaluation of their thermal stability. Energy dispersive X-ray (EDX) analysis was employed, using a Bruker AXS Microanalysis’ “XFlash 5010” instrument (Bruker, Billerica, MA, USA, EE.UU).

#### 4.3.3. Ion Exchange Capacity (IEC)

One of the key factors influencing fuel cell performance is proton transport through the PEM, which may be measured by a parameter known as ion exchange capacity (IEC). To determine this parameter, ionogel membranes were completely immersed in a 1 M HCl solution for 24 h. Subsequently, the ionogel membranes were removed from the HCl solution, washed with deionized water until neutral pH was reached, and placed in a 2 M NaCl solution for 24 h for Na^+^ ions to replace H^+^ ions. Finally, the ionogel membranes were removed and the resulting solution was titrated with 0.075 M NaOH by measuring the pH variation with a GLP21 PH-meter (Crison Instruments S.A., Barcelona, Spain) until the equivalence point was reached, determined by taking the first and second derivatives of the pH variation versus the volume of the titrating solution. The IEC values of ionogel membranes were calculated using the following equation:

(3) $$IEC\;\left(\frac{mmol}{g}\right)=\frac{V_{\mathrm{NaOH}}\times C_{\mathrm{NaOH}}}{W_{\mathrm{dry}}}$$

where *V*_NaOH_ is the volume of the titrant solution (L), *C*_NaOH_ (mmol/L) is the concentration of the titrant solution, titrated with a standard solution of oxalic acid, and *W*_dry_ is the dry weight of the ionogel membrane (g).

#### 4.3.4. Water Uptake

Another key indicator on the feasibility of ionogel membranes in fuel cells is water uptake, which is related to the transport of ions through them and thus to the ion exchange capacity (IEC). The method used to measure water uptake is relatively simple and has been described by other authors [39]. Once prepared, the ionogel membranes were weighed using an analytical balance and placed in deionized water for 24 h at room temperature. Afterwards, the water remaining on the surface of the ionogel membranes was removed with a tissue paper and membranes were reweighed. The equation used for the calculation of water uptake is as follows:

(4) $$\%\;Water\;uptake=\left(\frac{W_{\mathrm{wet}}-W_{\mathrm{dry}}}{W_{\mathrm{dry}}}\right)\times 100$$

where *W*_dry_ (g) is the weight of the dry membrane, before being introduced into the deionized water bath, and *W*_wet_ (g) is the weight of the membrane after the bath.

#### 4.3.5. Oxidative Stability

The oxidative stability of the membranes was evaluated by immersing ionogel membranes weighing 1.88 g in a solution of Fenton’s reagent (4 ppm Fe^2+^, added as Mohr’s salt + 3 wt% H_2_O_2_) at 60 °C. The ionogel membrane was weighed after preparation and placed in the Fenton’s solution at 60 °C for 24 h. After this, the membrane was rinsed with distilled water and placed in a vacuum oven at 40 °C for 2 h before weighing. This process was repeated in periods of 24 h, using fresh Fenton’s solution each time, for 7 days.

#### 4.3.6. Fourier Transform Infrared Spectroscopy (FTIR) and ^1^HNMR Proton Nuclear Magnetic Resonance

Fourier transform infrared (FTIR) spectra of the dried membranes were recorded on a Thermo Nicolet 5700 (Waltham, MA, USA), equipped with a DTGS KBr detector, in a spectral range from 4000 to 400 cm^−1^ and using a resolution of 4 cm^−1^.

The ^1^HNMR measurements of the different ionogel membranes were performed using a Bruker Avance Neo Nanobay 400 MHz with a spectral width of 12 ppm and tetrahydrofuran as a solvent.

### 4.4. MFC Studies

The experimental setup consists of reactors made from modified 250 mL glass bottles with cylindrical flanges, where the temperature was maintained at 25 °C. 

The membrane electrode assembly (MEA) was configurated with a PEM formulated like the ionogel membranes described in Section 4.2, the catalyst layer of the cathode was sprayed with platinum 60% nominally (Cymit quimica) with a loading of 0.5 mg/cm^2^ Nafion dispersion (D2021CS Alcohol based 1100 EW 20% weight, Ion Power, Munich, Germany) with a loading of 2.5 mg/cm^2^ to achieve a 3:1 ionomer to carbon ratio with 2-propanol (Cymit química) and 0.5 mL/cm^2^. The catalyst ink was submerged in an ultrasonic bath during 2 h before spraying into the PEM membrane. No sandwich was permitted due to the material of the membranes. The cathode was connected to the anode using a 1 kOhm resistor. The anode was composed of 100 g of graphite granules with a diameter of 3–5 mm and a graphite rod of 3.18 mm. Anode chambers contained 200 mL of wastewater and were sealed with a lip equipped with a sampling port, ensuring anaerobic conditions throughout the experiments. The MEA was securely fastened to the reactor flange using a round joint. All tests were conducted in batch mode using wastewater as the sole source of microorganism and fuel. Slurry wastewater (DQO = 2260 mg/L) was used as the fuel cell.

### 4.5. Statistical Analysis

Data were analyzed with PASW Statistics 28 for Windows (SPSS Inc., Chicago, IL, USA). A Student’s *t*-test, with a significance threshold set at *p* < 0.05, was performed to evaluate variations among the treatments analyzed. Values shown represent the mean ± standard deviation of three independent replicates for all analyses performed. Pearson’s correlation with a 95% confidence interval was used to determine bilateral correlations between membranes. Sigma Plot 12.5 was used to perform regression analyses using linear regression analysis. Confidence intervals with 95% confidence levels were established to accompany the estimated data.

## Figures and Tables

**Table 1:** Peak element vs. weight (%) for EDX spectra of the membranes M1 and M3, based on [N_8,8,8,1_^+^][Cl^−^], with IL concentration of 50% and 70%, respectively; M4 and M5, based on [N_8-10,8-10,8-10,1_^+^][Cl^−^] (Aliquat) and [N_8,8,8,1_^+^][Br^−^] at 70% of IL and 30% of PVC; and Nafion 212. Results recorded at room temperature and after 72 h of thermal analysis at 60 °C and 90 °C.

% Weight															
Peak Element	M1			M3			M4			M5			M CONTROL		
	25 °C	60 °C	90 °C	25 °C	60 °C	90 °C	25 °C	60 °C	90 °C	25 °C	60 °C	90 °C	25 °C	60 °C	90 °C
C K	73	85.2	81.5	85.6	76.43	79.2	85.24	78.57	77.25	72.83	75.38	77.79	23.11	23.51	21.23
N K	1.49	2.33	0	2.83	2.29	2.33	2.57	2.17	1.61	2.18	1.93	1.82	0	0	0
O K	1.21	0.31	6.58	0.24	0.36	6.07	0.36	0.54	4.7	0.25	0.59	3.32	8.06	6.34	6.49
S K	0	0	0.18	0	0	0	0	0	0	0	0	0	6.82	6.09	5.29
Mg K	0.09	0	0	0	0	0	0	0	0	0	0	0	0	0	0
Si K	0.12	0	0	0	0	0.12	0	0	0	0	0	0	0	0	0
Cl K	24.1	12.1	11.5	11.33	20.93	11.7	11.83	18.72	16.15	11.61	8.34	1.92	0	0	0
Br L	0	0	0	0	0		0	0	0	13.13	13.76	15.15	0	0	0
Ca K	0	0	0.3	0	0	0.58	0	0	0	0	0	0	0	0	0
F	0	0	0	0	0	0	0	0	0	0	0	0	62	64.05	67

**Table 2:** Membrane preparation of 70/30% [N_8-10,8-10,8-10,1_^+^][Cl^−^]/PVC.

PEM ID	Scale Factor	Total Weight (g)	IL Weight (g)	PVC Weight (g)
PEM 130	1.30	1.213	0.849	0.364
PEM 115	1.15	1.073	0.751	0.322
PEM 100	1.00	0.933	0.653	0.280
PEM 85	0.85	0.793	0.555	0.238
PEM 70	0.70	0.653	0.457	0.196

**Table 3:** Thickness of membranes based on 70/30% [N_8-10,8-10,8-10,1_^+^][Cl^−^]/PVC. Values are the mean ± standard deviation of three replicates.

PEM ID	Scale Factor	Total Weight (g)	Thickness (µm)
PEM 130	1.30	1.213	476.4 ± 10.2
PEM 115	1.15	1.073	425.1 ± 18.3
PEM 100	1.00	0.933	359.1 ± 5.7
PEM 85	0.85	0.793	323.2 ± 9.5
PEM 70	0.70	0.653	266.9 ± 21.7

**Table 4:** Effect of temperature on thermograms through the weight (%) of ionogel at 200 °C and the highest derivative temperature HDT (°C) in the thermograms.

Chemicals	200 °C Weight (%)	T (°C) HDT
[N_8,8,8,1_^+^][Cl^−^]	78.3	206.7
[N_8-10,8-10,8-10,1_^+^][Cl^−^]	78.8	205.9
[N_8,8,8,1_^+^][Br^−^]	93.0	230.0
PVC	100	281.5
50/50% [N_8,8,8,1_+][Cl^−^]/PVC	82.2	260.3
60/40% [N_8,8,8,1_^+^][Cl^−^]/PVC	83.9	274.5
70/30% [N_8,8,8,1_^+^][Cl^−^]/PVC	80.6	274.5
70/30% [N_8-10,8-10,8-10,1_^+^][Cl^−^]/PVC	81.8	334.3
70/30% [N_8,8,8,1_^+^][Br^−^]/PVC	83.6	280.5
Nafion 212	96.5	472.2

**Table 5:** Ion exchange capacity of the different ionogel membranes mixtures prepared. Values are the mean ± standard deviation of three replicates. M1: 50/50% [N_8,8,8,1_^+^][Cl^−^]/PVC. M2: 60/40% [N_8,8,8,1_^+^][Cl^−^]/PVC. M3: 70/30% [N_8,8,8,1_^+^][Cl^−^]/PVC. M4: 70/30% [N_8-10,8-10,8-10,1_^+^][Cl^−^]/PVC. M5: 70/30% [N_8,8,8,1_^+^][Br^−^]/PVC.

Membrane	Ion Exchange Capacity (mmol/g)
Nafion 212	0.865 ± 0.013
M1	0.022 ± 0.002
M2	0.028 ± 0.008
M3	0.015 ± 0.003
M4	0.018 ± 0.001
M5	0.042 ± 0.008

**Table 6:** Maximum power density. Values are the mean ± standard deviation of three replicates. Values are shown for 50/50% [N_8,8,8,1_^+^][Cl^−^]/PVC; 60/40% [N_8,8,8,1_^+^][Cl^−^]/PVC; 70/30% [N_8-10,8-10,8-10,1_^+^][Cl^−^]/PVC; and 70/30% [N_8,8,8,1_^+^][Br^−^]/PVC.

MFC	Maximum Power Density (mW/m^2^)	% vs. Control
50/50% [N_8,8,8,1_^+^][Cl^−^]/PVC	104.02 ± 8.03	38.89
60/40% [N_8,8,8,1_^+^][Cl^−^]/PVC	147.42 ± 1.79	55.11
70/30%[N_8-10,8-10,8-10,1_^+^][Cl^−^]/PVC	87.04 ± 2.67	32.54
70/30% [N_8,8,8,1_^+^][Br^−^]/PVC	116.7 ± 3.15	43.63
Nafion 212	267.49 ± 29.84	100

**Table 7:** Relevant information about ionogel membranes mixtures prepared.

Membrane Code	Composition (%)				Total Weight (g)	Circular Flat Glass Area (cm^2^)	Analyses Performed
	PVC	[N_8,8,8,1_^+^][Cl^−^]	[N_8-10,8-10,8-10,1_^+^][Cl^−^]	[N_8,8,8,1_^+^][Br^−^]			
M1	50%	50%	-	-	0.49	9.90	TGA, Thermal stability test, SEM-EDX
					1.88	38.07	IEC, water uptake, Fenton, FTIR, ^1^HNMR
M2	40%	60%	-	-	0.49	9.90	TGA, Thermal stability test, SEM-EDX
					1.88	38.07	IEC, water uptake, Fenton, FTIR, ^1^HNMR
M3	30%	70%	-	-	0.49	9.90	TGA, Thermal stability test, SEM-EDX
					1.88	38.07	IEC, water uptake, Fenton, FTIR, ^1^HNMR
M4	30%	-	70%	-	0.49	9.90	TGA, Thermal stability test, SEM-EDX
					1.88	38.07	IEC, water uptake, Fenton, FTIR, ^1^HNMR
M5	30%	-	-	70%	0.49	9.90	TGA, Thermal stability test, SEM-EDX
					1.88	38.07	IEC, water uptake, Fenton, FTIR, ^1^HNMR

## Data Availability

Data are available under reasonable request.
